# Isolation, Characterization, and Pharmaceutical Applications of an Exopolysaccharide from *Aerococcus Uriaeequi*

**DOI:** 10.3390/md16090337

**Published:** 2018-09-16

**Authors:** Chunlei Wang, Qiuping Fan, Xiaofei Zhang, Xiaoping Lu, Yanrui Xu, Wenxing Zhu, Jie Zhang, Wen Hao, Lujiang Hao

**Affiliations:** 1 State Key Laboratory of Biobased Material and Green Papermaking, Qilu University of Technology, Shandong Academy of Sciences, Jinan 250353, China; chunlei_wang79@163.com (C.W.); fanqiuping1990@163.com (Q.F.); xiaofei_305@163.com (X.Z.); 15269213716@163.com (X.L.); 15269211209@163.com (Y.X.); zhwenxing@163.com (W.Z.); zhangjie@qlu.edu.cn (Z.J.); 2 Qingdao Municipal Center for Disease Control & Prevention, Qingdao 266033, China; cdchaowen@126.com; 3 Qingdao Institute of Preventive Medicine, Qingdao 266033, China

**Keywords:** marine bacteria, EPS-A, structural analysis, sewage flocculation, oxidation resistance

## Abstract

Many marine bacteria secrete exopolysaccharides (EPSs), which are made up of a substantial component of the macro-molecules surrounding cells. Recently, the wide demand for EPSs for food, cosmetics, pharmaceutical and other applications has led to great interest in them. In this study, an EPS produced by marine bacteria *Aerococcus uriaeequi* HZ strains (EPS-A) was isolated and purified to examine its structure and biological function. The molecular weight of EPS-A analyzed by high-performance liquid gel filtration chromatography (HPGFC) is found to have a number average of 2.22 × 10^5^ and weight average of 2.84 × 10^5^, respectively. High-performance liquid chromatography (HPLC) and Fourier-transform–infrared (FT–IR) analysis indicate that EPS-A was a polysaccharide composed of glucose and a little mannose. In addition, the flocculating rate of sewage of EPS-A was 79.90%. The hygroscopicity studies showed that hygroscopicity of EPS-A was higher than chitosan but lower than that of sodium hyaluronate. The moisture retention of EPS-A showed similar retention activity to both chitosan and sodium hyaluronate. EPS-A also can scavenge free radicals including both OH• free radical and O_2_•^−^ free radical and the activity to O_2_•^−^ free radical is similar to vitamin C. Safety assessment on mice indicated that the EPS-A is safe for external use and oral administration. EPS-A has great potential for applications in medicine due to its characteristics mentioned above.

## 1. Introduction

Microbial exopolysaccharides are critical for the biofilm formation, which is involved in the protection of bacteria against a harmful environment and in the adherence. The EPS layers with high viscosity are formed by accumulating various types of polymeric substances. They tend to be hygroscopic and aerophytic bacteria, and often contain more water than the surrounding environment [1]. It is known that certain marine bacterial polysaccharides have features such as hygroscopicity and moisture resistance, ability to scavenge free radicals, oxidation resistance, and the adsorption of heavy metal ions. The EPS from *Pseudoalteromonas* SM20310 enables the strain to adapt to the environments such as low temperature, high salt concentration, and freeze–thaw cycles. In addition to its functions in the strain, the EPS also obviously increased the tolerance of *Escherichia coli* to repeated freeze–thaw cycles [2]. Many studies indicate that the biological activities of polysaccharides are related to their structural features, including molecular weight and concentration, the compositions of sugar, branch structures, and type of glycosyl linkage [3].

Polysaccharides and their derivatives have been widely used in industries, such as food process, pharmaceutics, cosmetic, and health care. For instance, polysaccharides extracted from algae have been applied as the excipients in cosmetic formulae because of their high bonding, gelling, and viscosity-increasing properties [4]. Moreover, the polysaccharide from *Morchella conica* (Fungi, Ascomycota) may act as a powerful immunomodulatory agent by modulating nitric oxide production in macrophages and subserve splenocytes proliferation [5]. EPSs can be readily prepared in the laboratory by fermentation [6]. Previous experiments proved that the yield of EPS from the submerged fermentation of Chinese truffle *Tuber sinense* (Fungi, Ascomycota) can reach 1.59 g/L under predicted optimal conditions [7]. *Pseudoalteromonas* SM20310 (Bacteria, Proteobacteria) screened from 110 Arctic sea ice can produce EPS with of 0.57 g/L [2]. The bacterial strain CAM025 from *Pseudoalteromonas* isolated from Antarctic yield *ca* 100 mg EPS per gram dry weight of cells of EPS at −2 °C and at 10 °C [8].

In this study, a marine bacterium *Aerococcus uriaeequi* HZ with the production of EPSs was screened from abalone aquaculture environments. In order to study the production of the EPSs produced by *A. uriaeequi* HZ, the strain was cultured and an EPS was extracted, isolated, and purified (names EPS-A). We also analyze the structure and composition of EPS-A by Fourier-transform–infrared (FT–IR) spectrometry and high-performance gel filtration chromatography (HPGFC). Finally, its flocculation properties, moisture-absorption and retention abilities and antioxidant activity were studied to explore its potential application in biotechnology.

## 2. Results

### 2.1. Extraction and Purification of Microbial Exopolysaccharide (EPS-A)

**Figure 1:**
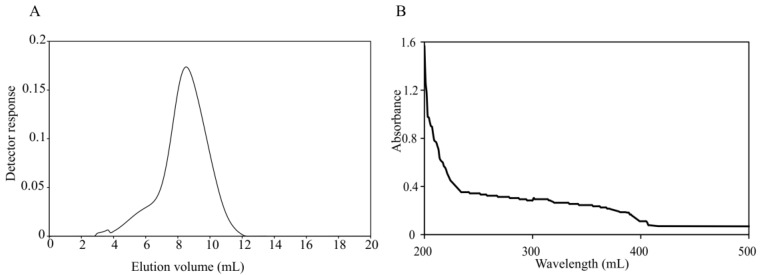
Purification of EPS-A. (**A**) Mw distribution of EPS-A determination by high-performance liquid gel permeation chromatography (HPGFC). HPGFC was performed using a Shodex SB-806HQ column in 0.2 M NaCl solution with 0.5 mL/min flow rate. (**B**) Ultraviolet (UV)-visible spectrum of EPS-A. The absorbance from 200–500 nm was measured in H_2_O at room temperature.

EPSs from bacteria have been widely used as anticoagulant, antithrombotic, immunomodulation, anticancer agents and as bioflocculants in areas such as pharmacological, nutraceutical, functional food, cosmeceutical and herbicides [9]. Many marine bacteria secrete EPSs as a strategy for growth, adherence, and to survive under harmful conditions [10]. *A. uriaeequi* HZ strain was isolated from the Yellow Sea of China. To analyze if the bacteria can produce EPSs, *A. uriaeequi* was fermented and the EPS-A was isolated by Diethylaminoethano (DEAE) ion exchange chromatography and gel filtration chromatography. The freeze-dried product was yellow-white, water-soluble powder. HPGFC results showed that the purified product forms a single and symmetric peak, which indicated that EPS-A was purified with high quality. In order to avoid the presence of protein on the production of extracellular polysaccharides, the products were scanned by a ultraviolet (UV)-visible spectrophotometer. EPS-A did not show absorption at 260 nm and 280 nm in the UV spectrum (Figure 1B), which suggested that the nucleic acid and protein was in the absence [11], and the total sugar content of the EPS is ca 2.34 g/L. The molecular weight of EPS-A was further measured by HPGFC, and the molecular distribution of HPGPC of EPS-A is shown in Figure 1A. The result showed that EPS-A is the sugar with the number average 2.22 × 10^5^ and weight average 2.84 × 10^5^, respectively. The distribution coefficient was 1.28, indicating the small coefficient dispersion of EPS-A (Table 1).

### 2.2. Monosaccharide Composition Analysis of EPS-A

**Figure 2:**
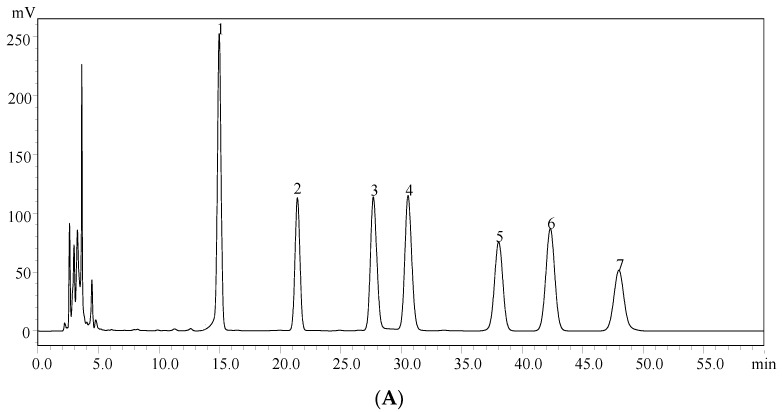

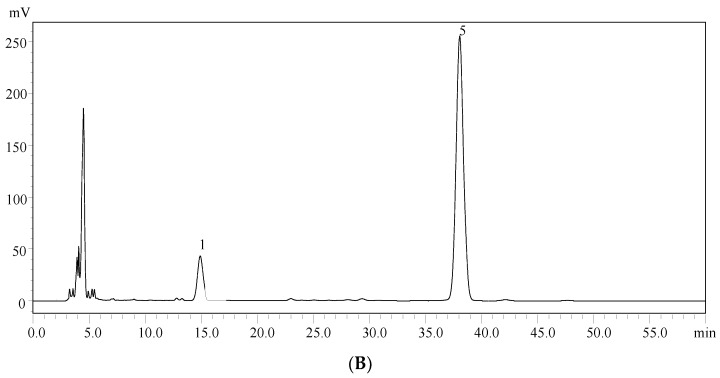
High-performance liquid chromatography (HPLC) chromatograms of seven PMP-labeled standard monosaccharides (**A**) and PMP-labeled monosaccharides released from EPS-A (**B**). Peaks: 1. D-Mannose; 2. D-Rhamnose; 3. D-Glucuronic acid; 4. D-Galacturonic acid; 5. D-Glucose; 6. D-Galactose;7. d-Xylose.

Monosaccharide composition analysis is used to determine the identities and quantities of the various monosaccharides in the carbohydrates and glycoproteins. The information can be used to analyze the structure of carbohydrates and play an important role in quantification. The monosaccharide composition of EPS-A was identified by high-performance liquid chromatography (HPLC). EPS-A was hydrolyzed by sulfuric acid and derivatized with 1-phenyl-3-methyl-5-pyrazolone (PMP). As shown in Figure 2, two kinds of monosaccharide were found, D-mannose and D-glucose, accounting for 10.71% and 66.99%, respectively. As compared with PMP-labeled standard monosaccharides (Figure 2A), the molar ratio of D-mannose and D-glucose of EPS-A was 1:9.65 (Figure 2B). This result was different from an exopolysaccharide produced by *Bifidobacterium animalis* with Mw = 21.3 kDa, which was composed of the monosaccharides including arabinose, galactose, glucose, mannose, and rhamnose [12].

### 2.3. Fourier-Transform–Infrared (FT–IR) Analysis

FT–IR spectroscopy is a widely used method that shows the infra-red-light absorption by molecular bonds at a given wavelength [13]. The method can analyze the polysaccharide structure, such as monosaccharide types, glucosidic bonds, and functional groups, by investigating the vibrations of molecules and polar bonds between atoms [14,15,16].

**Figure 3:**
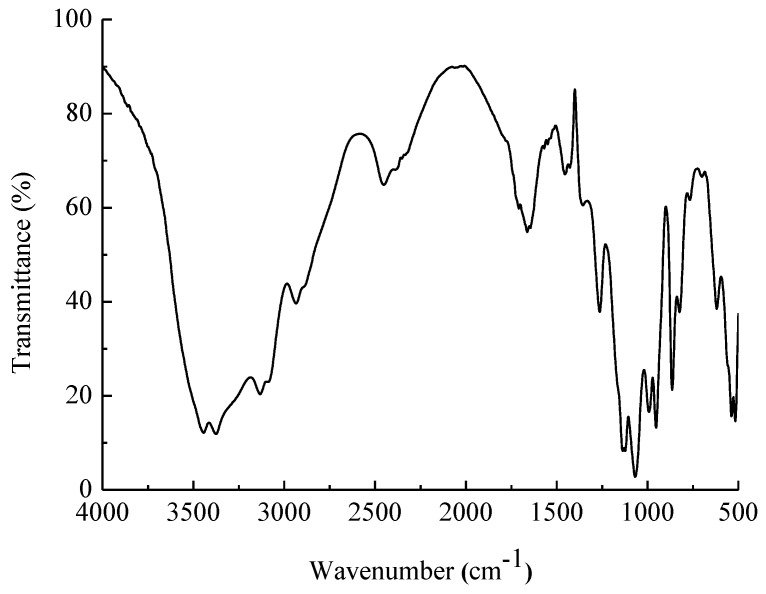
FT-IR spectra of EPS-A. Dried polysaccharides were ground and pelletized with KBr. Ultraviolet-visible spectrum of EPS-A was recorded with a spectrophotometer from 500–4000 cm.

EPS-A had polysaccharide characteristic of a broad absorption peak in 3200~3650 cm^−1^, which was the O–H stretching vibration peak (Figure 3). The peak at 2887.4 cm^−1^ and 2935.6 cm^−1^ were -C–H stretching vibration of –CH_3_ and –CH_2_, respectively. Around 1100 cm^−1^, there are three absorption peaks, indicating the presence of monosaccharides in pyran form in the extracellular polysaccharide. The characteristic absorption peak at 817 cm^−1^ confirmed the presence of α-D-mannopyranose [17,18]. The structure of the infrared spectrometry confirmed the measurement results of the monosaccharide composition.

### 2.4. Bio-Flocculating Activity

**Figure 4:**
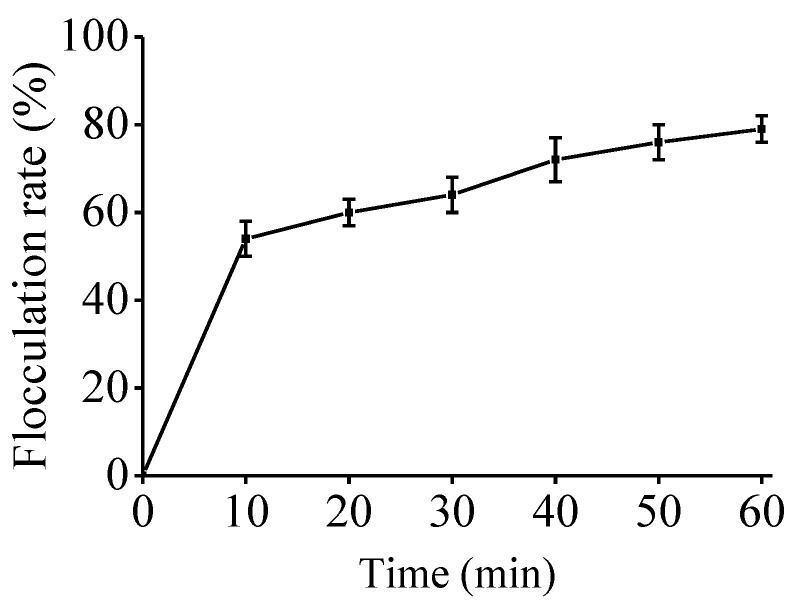
Sewage flocculation assay of EPS-A. 0.2 g EPS was added l00 mL sewage and incubated for 1 h. The function of sewage flocculation was measured at 550 nm by a spectrophotometer.

Flocculation technology as a kind of effective and quick method is used for wastewater treatment. Bio-flocculation is a dynamic process owing to the ability to form extracellular polymers by living cells. Recently, microbial flocculants have been widely used, which are innocuous and biodegradable. The flocculating activity of an EPS by *Bacillus thuringiensis* is 80.4% [19]. An EPS produced by a marine dinoflagellate *Gyrodinium impudicum* (Chromista, Dinophyceae) had >90% flocculating activity [20]. In this study, the flocculating rate was as high as 79.90% (Figure 4), With the increase of concentration, the flocculation ability of EPS-A gradually increased. The results show that EPS-A could be an excellent candidate as a kind of sewage treatment agent that is non-toxic, harmless, no secondary pollution.

### 2.5. Moisture Absorption and Retention Properties EPS-A

EPSs are hygroscopic and therefore have the ability to maintain a high water content in the microenvironment [21]. The hygroscopic property has been widely used in the food industry [22]. The hygroscopicity of EPS-A was investigated together with chitosan and sodium hyaluronate. The experimental results showed that EPS-A moisture absorption was significantly higher than that of chitosan but less than that of sodium hyaluronate.

**Figure 5:**
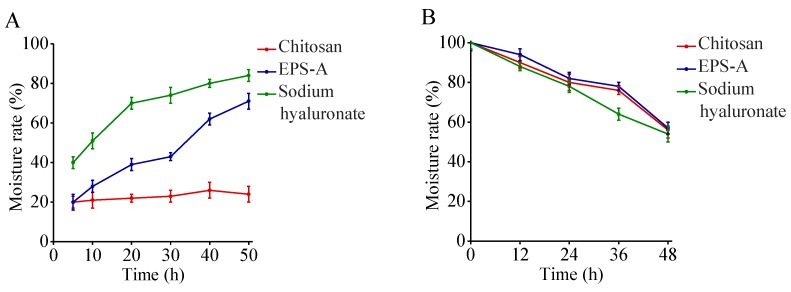
Moisture-absorption and retention activity of EPS-A. (**A**) Hygroscopic activity assay of EPS-A. Hygroscopic activity of EPS-A was determined by measuring the increased weight of absorbing H_2_O by EPS-A. Chitosan and sodium hyaluronate were used as controls. The value obtained at 50 h by sodium hyaluronate was set 100%. (**B**) Moisture retention activity assay of EPS-A. Moisture retention of EPS-A was determined by measuring the reserved weight of H_2_O by EPS-A. Chitosan and sodium hyaluronate were used as controls. And the value at the beginning was set 100%.

The moisture retention ability of EPSs plays an important role in cosmetics and clinical medicine [23]. The moisture retention of EPS-A was studied and compared with that of chitosan and sodium hyaluronate. The results showed EPS-A has similar moisture-absorption abilities as that of chitosan and sodium hyaluronate (Figure 5). The moisture absorption and retention properties make EPS-A valuable in the food industry, clinical medicine, and cosmetics.

### 2.6. Removal Results on Free Radicals

**Figure 6:**
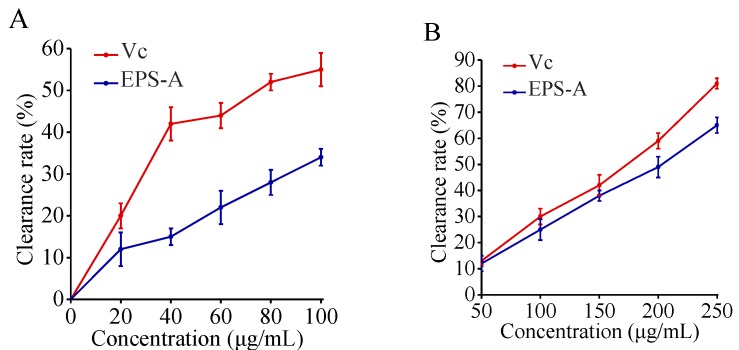
Free radical scavenging activity of EPS-A. **A** OH• free radical scavenging activity of EPS-A. The scavenging activity to OH• of different concentration EPS was determined by removing OH• generated by FeSO_4_ and H_2_O_2_. Vitamin C was used as a control and the activity of 100 µg/mL vitamin C was set 100%. **B** O_2_•^−^ free radical scavenging activity of EPS-A. The scavenging activity to O_2_•^−^ of different concentration EPS-A was determined by removing O_2_•^−^ generated from pyrogallol. Vitamin C was used as a control and the activity of 100 µg/mL Vitamin C was set 100%.

The oxygen free radicals such as superoxide radical anion (O_2_•^−^) and hydroxyl radical (OH•) are highly potent oxidants that can react with the biomacromolecules in cells and are related to mutagenesis and carcinogenesis. The antioxidant properties of polysaccharides from algae, plant, fungi, and prokaryotes have been studied for their antioxidant properties as potential therapeutic [24]. For example, *Dixoniella grisea (formerly Rhodella reticulata, Rhodophyta)* EPS had a stronger ability against O_2_•^−^ than α-tocopherol and the crude polysaccharides were twice as strong as α-tocopherol [25]. Natural EPS from *Brevibacterium otitidis* (Bacteria, Actinobacteria) also possessed a strong free radicals scavenging effect, which can be comparable to vitamin C [26]. In this study, OH• free radical and super oxygen anion O_2_•^−^ scavenging activity of EPS-A were analyzed (Figure 6). The results showed that the scavenging effect of EPS-A on OH• radicals increased with increasing concentration of EPS-A. when EPS-A concentration reached 100 μg/mL, the clearance rate reached 45.65%, which is lower than the value of Vitamin C (Vc). Scavenging results of EPS-A on O_2_•^−^ also showed a significant dose–effect relationship. When the concentration of EPS-A reached 250 μg/mL, the clearance rate was 67.31%, which is near to Vc. EPS-A showed similar clearance rate to both superoxide radical anion and hydroxyl radical, even the rate is lower than Vc. Then EPS-A may also be explored as a novel potential antioxidant. The hydroxyl radical scavenging activities of EPSs were attributed to various mechanisms. One possibility was that EPSs could absorb radicals and terminate the radical reaction [27].

### 2.7. Safety Assessment of EPS-A

**Figure 7:**
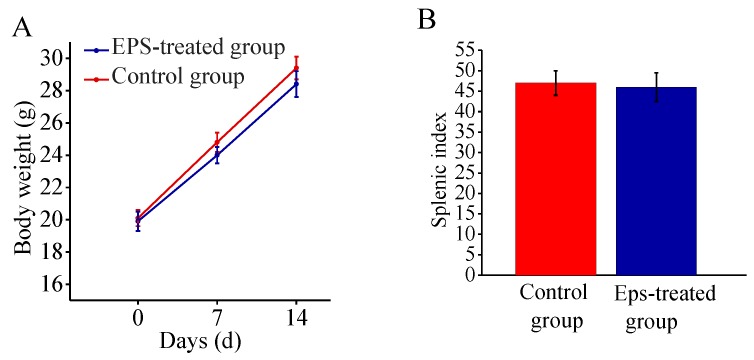
Effect of EPS-A on the body weight (**A**) and splenic indices (**B**) of mice in a 14-day feeding test.

An acute toxicity test on mice was performed to assess the safety of the EPS-A. The mice were orally administered a dose of 5000 mg/kg of the EPS-A. No mice died in either the treated or the control group in the 14-day test period. In both groups, the mean body weight of mice increased gradually and did not show a significant difference during growth, and at the end of the test (Figure 7A). there was also no substantial difference of the splenic indices between treated mice and the untreated controls (Figure 7B). The acute toxicity test indicated that EPS-A is safe for usage.

## 3. Discussion

Microbial EPSs have attracted great interest among scientists because of their wide potential applications spanning areas such as health (pharmaceuticals and medicine), industry (cosmetics, textile, dairy etc.), and environment (flocculation, remediation, etc.) [28]. However, only a few microbial EPSs have been used commercially. Among them, microbial dextran could be considered as the first example used in food and pharmaceutical industries [24]. Their production costs are the main constraints to full commercialization, especially the substrate cost and the cost of purification processing [29]. EPSs are of considerable value in the removal of pollutants from wastewater, in the dewatering of activated sludge, and in bioflocculation and settling [30]. Flocculation activity >75% was obtained using very low concentrations of EPSs from *Bacillus*, and our results showed that EPS-A showed activity of 79.90%.

**Figure 8:**
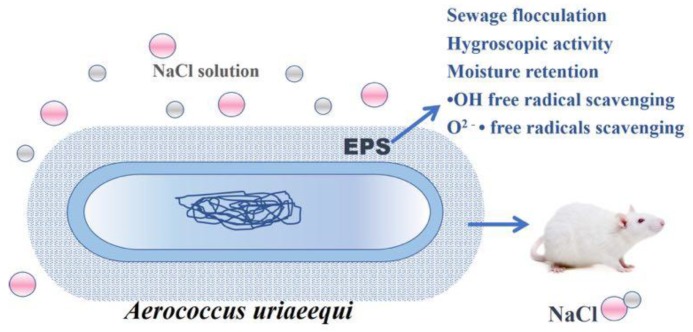
Schematic diagram of EPS-A with the potential advantages (e.g., Sewage flocculation, Hygroscopic activity, Moisture retention, •OH free radical scavenging, O^2−^ • free radicals scavenging). The safety assessment will be performed on mice.

In this study, EPS-A showed good moisture-absorption and retention ability. The moisture-absorption ability of EPS-A was between chitosan and sodium hyaluronate; and its moisture-retention ability was comparable with chitosan and sodium hyaluronate. Similarly, Sun et al. [31] found the moisture-absorption ability of extracellular polysaccharide produced by an Arctic marine bacteria was higher than that of chitosan but less than that of sodium hyaluronate. Moreover, the moisture-retention ability was higher than that of chitosan and sodium alginate. To my knowledge, few studies are able to clarify this mechanism of extracellular polysaccharides. Chen’s group [32] reported that the moisture-absorption and retention ability of Carboxymethyl chitosans (CM-chitosan) containing different subrogation points was related to the active sites of 6-carboxymethyl in the molecular structure. Moreover, the carboxymethylation of N sites promote moisture-absorption and retention ability which increase with its molecular weight. The extracellular polysaccharides of the Arctic marine bacteria are mainly composed of N-acetyl glucosamine, glucuronic acid, mannose, medium galactose, and fucose, as well as a small amount of rhamnose and glucose. In this study, the EPS-A is proved to consist of glucose and a small amount of mannose. It is speculated that the monosaccharide composition of the bacterial extracellular polysaccharide and the molecular weight of the extracellular polysaccharide have a great relationship with the moisture-absorption and retention ability, and the mechanism of the difference in moisture-absorption and retention ability need to be revealed in the later period. In addition, moisture-retaining and absorbing bio-materials have been extensively used in cosmetic, food, pharmaceutical and other industries [31]. The moisture-retention ability of EPS-A reveals its great potential as a wound dressing and moisturizing ingredient [33]. Also, EPS-A showed its antioxidant activity as Vc. The antioxidant properties of EPSs are important functions in maintaining human health and preventing disease. For example, an EPS from *Pleurotus salmoneo-stramineus* (Fungi, Basidiomycota) with antioxidant activity represented a surprising antitumor capability against colon cancer [34]. In summary, our study showed that EPS-A is a promising biomaterial for food, cosmetics and medical applications (Figure 8).

## 4. Materials and Methods

### 4.1. Cell Culture

Marine bacterium *A. uriaeequi* HZ [35] strain was isolated from abalone breeding environment in Rongcheng, Shandong Province, China, and cultivated on Zobell 2216E solid medium containing 5.0 g/L peptone, 1.0 g/L yeast extract, 0.01 g/L sodium phosphate, and 35 g/L bay salt with pH 7.6~7.8 at 25 °C. The medium with 30 g/L sucrose, 2.5 g/L beef extract, 30 g/L bay salt, and 0.01 g/L sodium phosphate was used for fermentation study to produce EPS-A.

### 4.2. Production and Purification of EPS-A

*A. uriaeequi* HZ was cultured in the flask at 25 °C for 8 h, then 10% seed culture was inoculated in fermentation flasks and incubated on a shaker with 230 rpm at 25 °C for 35 h. The fermentation broth was centrifuged at 4000 rpm for 20 min. Ethanol was added to the supernatants to reach the final concentration of 60% (v/v) and precipitated overnight at 4 °C [36]. The precipitates were centrifuged at 4000 rpm for 20 min to remove the supernatants. The pellets were dissolved in water and mixed with Savage reagent (chloroform: n-butanol = 5:1) at a 1:4 volume ratio. The solution was mixed sufficiently and centrifuged to remove organic solvents and denatured protein [37]. The total sugar content of the EPS was determined by the phenol-sulfuric acid method [35].

The EPS in deionized water was further purified by using DEAE-52 anion-exchange chromatography with a 1.6 cm × 30 cm column. The samples were eluted at a flow rate of 60 mL/h with a linear gradient of 0 to 1 M NaCl solution in the system. The EPS fraction was further purified using gel filtration chromatography (Sepharose 4B) on a column (1.6 cm × 100 cm), which was eluted at a flow rate of 12 mL/h with 0.1 M NaCl solution [2]. Then the purified EPS was dialyzed by deionized water using a selective semi-permeable membrane (8000~14,400 da). The polysaccharide was freeze-dried and stored at 4 °C until analysis [38].

### 4.3. Molecular Weight Determination of EPS-A

**Figure 9:**
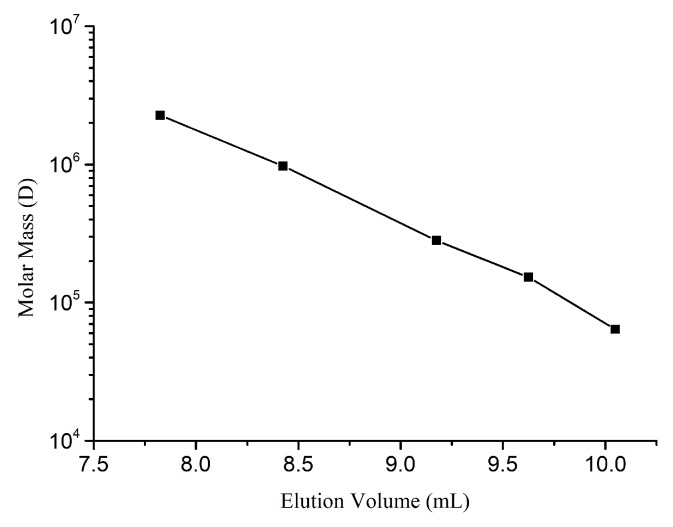
Standard curve of HPGPC-PSS series of standard samples.

The molecular weight of EPS-A was determined by gel filtration chromatography [39]. HPGFC was used for analysis using a Shodex SB-806HQ column (0.8 cm× 30 cm, Agilent, Santa Clara, CA, USA). The mobile phase included 0.2 M NaCl in H_2_O. The system was run at a flow rate of 0.5 mL/min at 35 °C. 100 μL standard sample (Table 2) was injected into the liquid chromatograph. The chromatogram was recorded, and the universal correction and the linear regression equation was performed and calculated by Gel Permeation Chromatography (GPC) software (A.02.01, Agilent, Santa Clara, CA, USA). The EPS-A was determined in the same method as above. The standard curve was shown in Figure 9. The k value of the reference substance was 0.0006, and the α value was 0.75. The molecular weight and molecular weight distribution of EPS-A were calculated by GPC software.

### 4.4. Hydrolysis and Derivatization of EPS-A

Twenty mg EPS-A was mixed with the 10 mL sulfuric acid solution (1 Mol/L) and incubated at 100 °C for 8 h for hydrolysis. After centrifugation, the hydrolysate at 10,000 r/min for 10 min, 4.5 mL of supernatant was pipetted and neutralized to pH 7 with 2 mol/L NaOH, and the neutralized solution was made up to 10 mL. PMP derivatization of monosaccharides was carried out as described previously with proper modification [40,41]. Briefly, 50 μL neutralized solution, 50 μL PMP-methanol solution (0.5 mol/L) and 50 μL NaOH solution (0.3 mol/L) were mixed in a 1.5 mL tube, which was placed in a constant temperature water bath at 70 °C for 30 min. After cooling to room temperature, 50 μL of HCl solution was added to the mixture for neutralization, and 100 μL of ultrapure water was added for dilution. The mixture was extracted 3 times with chloroform, and filtered by a 0.45-micron filter before HPLC. The 2 mmol/L standard solution of mannose, rhamnose, glucuronic acid, galacturonic acid glucose, galactose, and xylose was derivatized by the same method. All the assays were independently conducted in triplicate.

### 4.5. High-Performance Liquid Chromatography (HPLC) Analysis of the Monosaccharide Composition

An HPLC (DGU-20A, Shimadzu, Kyoto, Japan) equipped with a column InertSustain (4.6 mm × 250 mm, Shimadzu, Kyoto, Japan) was used for identification and quantification of the monosaccharide composition in EPS-A, The mobile phase was 80% ammonium acetate solution and 20% acetonitrile at a flow rate of 1.0 mL/min, and the column temperature was at 30 °C. The EPS-A was detected by UV detector at 245 nm [27,41].

### 4.6. Ultraviolet (UV)-Visible and FT–IR Spectroscopy

A UV-2450 spectrophotometer (Shimadzu, Kyoto, Japan) was used to record the ultraviolet-visible spectrum of EPS-A. A Fourier-transform–infrared spectrophotometer (PerkinElmer, Norwalk, CT, USA) was used to record FT-IR spectra of the samples [42].

### 4.7. Sewage Flocculation Analysis of EPS-A

The sewage in this study was collected from the sewage treatment station at Qilu University of technology; 0.2 g EPS-A was added to 100 mL of sewage in the 250 mL flask. The mixture was left to stand for l h. Absorbance was measured with a spectrophotometer at 550 nm. The original sewage photogenic liquid absorbance D_γ_ value was illustrated by the A1, the processed wastewater photogenic liquid absorbance D_γ_ value was illustrated by B1. The flocculation effect is in the flocculating rate:

Flocculating rate (%) = (A1 − B1)/A1 × 100%

### 4.8. Hygroscopic Activity of EPS-A

Flasks containing 0.1g EPS-A was placed in the sealed dryers with saturated NaCl or CaCl_2_ solution, respectively. The relative humidity in the two dryers is 73% and 32% respectively. After the preset time, the sample was weighted and hygroscopic rate was calculated. Chitosan and sodium hyaluronate were used as controls. The hygroscopic rate was calculated based on:

Hygroscopic rate (%) = (increased weight/original weight) × 100%

### 4.9. Moisture Retention Determination of EPS-A

Flasks containing 0.1 g EPS-A with 0.5 g of distilled water were incubated in the sealed dryers with a saturated CaCl_2_ solution or silica gel, respectively. The relative humidity in the two dryers is 32% and 0%. After the preset time, the sample was weighted and hygroscopic rate was calculated. Chitosan and sodium hyaluronate were used as controls. Moisture retention was calculated based on:

Moisture retention (%) = (weight of water after incubation/original weight of water) × 100%

### 4.10. OH• Free Radical Scavenging Activity 

The free radical scavenging activity of OH• was analyzed using Fenton’s reaction [43]. The hydroxyl radical was generated in a mixture of 1.0 mL 1,10-phenanthroline (5 mM), 1.0 mL sodium phosphate buffer (0.05 M, pH 7.4), 0.5 mL FeSO_4_ (7.5 mM) and 0.5 mL H_2_O_2_ (3%, *v*/*v*). A series of concentrations of EPS-A were added to the solution and reacted at 37 °C for 30 min. The absorbance was recorded at 510 nm. H_2_O and Vitamin C (Vc) were used as the blank and positive control, respectively. Clearance calculation formula was:

Clearance rate (%) = [A_0_ − (A_x_ − A_x0_) ]/A_0_ × 100%

where ΔA_0_ and ΔA denote the absorbance of the blank solution and the absorbance of the solution after addition of the EPS, respectively. A_x0_ was the absorption of the background of the polysaccharide solution.

### 4.11. O_2_•^−^ Free Radicals Scavenging Activity

The in vitro O_2_•^−^-scavenging activity of EPS-A was measured using the pyrogallol method [44]. Briefly, Reagents were added into a cuvette in the following order: 10 μL pyrogallol (3 mM), 80 μL NaOH (4 mM), 10 μL EPS-A, and 900 μL luminol (with a concentration of 0.1 mM in sodium carbonate buffer, pH = 10.2) and incubated in a water bath at 25 °C. A series of reactions with a final different concentration of EPS-A were set up and absorbance was recorded at 325 nm. Vc group was treated as control. The clearance calculation formula was:

Clearance rate (%) = (ΔA_0_ − ΔA)/(ΔA_0_) × 100%

where ΔA_0_ and ΔA denote the self-oxidation rate and the self-oxidation rate after adding EPS-A, respectively.

### 4.12. Safety Assessment of EPS-A

Acute oral toxicity of the EPS-A was analyzed following both the Good Laboratory Practice Standards manual and the Organization for Economic Cooperation and Development (OECD) Guidelines for Acute Toxicity of Chemicals no. 420 as previous reported [31]. KM (Kunming) female mice (8 weeks old, weight between 19 and 21 g) were obtained from the Experimental Animal Center of Shandong University (Jinan, China). All of the experimental protocols were approved by the Experimental Animal Ethic Committee of Qilu University of Technology, Shandong, China (Animal Experimental Ethical Inspection Protocol No. 201605004, 16 May 2016).

## 5. Conclusions

In this study, an exopolysaccharide from *Aerococcus uriaeequi* (EPS-A) was purified with the number average molecular mass of E 2.22 × 10^5^ g/mol and the weight average molecular mass of 2.84 × 10^5^ g/mol. EPS-A was mainly composed of glucose and mannose with β-configurations. EPS-A showed wastewater flocculation with 79.90 % activity. The sugar can also scavenge hydroxyl free radical in a clear dose–effect relationship. When the EPS-A concentration reached 100 μg/mL, the clearance rate reached 45.65% to •OH. When the concentration was 250 μg/mL, the clearance rate reached 67.31% to O_2_^−^ •. Finally, EPS-A also showed moisture-absorption and retention properties that can be compared with sodium hyaluronate and chitosan.

## Figures and Tables

**Table 1:** The yield and molecular weight distribution of microbial exopolysaccharide (EPS-A).

Total Sugar Content (g/L)	Number Average Molecular Mass (g/mol)	Weight Average Molecular Mass (g/mol)	Distribution Coefficient
2.34	2.22 × 10^5^	2.84 × 10^5^	1.28

**Table 2:** Standard samples of the Polystyrene sulfate (PSS).

Standard Samples LOT	MW (g/mol)	Weight (mg)
PSS929n	63,900	10.1
PSS8065n	152,000	10.2
PSS13092	282,000	10.1
PSS14052	976,000	10.0
PSS9304-4	2,260,000	10.0

## Abbreviations

EPS	exopolysaccharide
EPS-A	exopolysaccharide from *Aerococcus uriaeequi*
FT–IR	Fourier-transform–infrared spectrometry
HPGFC	high performance gel permeation chromatography

## References

[1] Helm R.F., Huang Z., Edwards D., Leeson H., Peery W., Potts M. (2000). Structural characterization of the released polysaccharide of desiccation-tolerant Nostoc commune DRH-1. J. Bacteriol..

[2] Liu S.B., Chen X.L., He H.L., Zhang X.Y., Xie B.B., Yu Y., Chen B., Zhou B.C., Zhang Y.Z. (2013). Structure and ecological roles of a novel exopolysaccharide from the arctic sea ice bacterium *Pseudoalteromonas* sp. Strain SM20310. Appl. Environ. Microbiol..

[3] He J., Zhang A., Ru Q., Dong D., Sun P. (2014). Structural characterization of a water-soluble polysaccharide from the fruiting bodies of *Agaricus bisporus*. Int. J. Mol. Sci..

[4] Kim S.K., Ravichandran Y.D., Khan S.B., Kim Y.T. (2008). Prospective of the cosmeceuticals derived from marine organisms. Biotechnol. Bioprocess Eng..

[5] Su C.A., Xu X.Y., Liu D.Y., Wu M., Zeng F.Q., Zeng M.Y., Wei W., Jiang N., Luo X. (2013). Isolation and characterization of exopolysaccharide with immunomodu-latory activity from fermentation broth of Morchella conica. DARU J. Pharm. Sci..

[6] lamas I.L., Mata J.A., Tallon R., Bressollier P., Urdaci M.C., Quesada E., Béjar V. (2010). Characterization of the exopolysaccharide produced by *Salipiger mucosus* A3(T), a halophilic species belonging to the alphaproteobacteria, isolated on the spanish mediterranean seaboard. Mar. Drugs.

[7] Liu R.S., Li D.S., Li H.M., Tang Y.J. (2008). Response surface modeling the significance of nitrogen source on the cell growth and Tuber polysaccharides production by submerged cultivation of Chinese truffle Tuber sinense. Process Biochem..

[8] Mancuso Nichols C.A., Garon S., Bowman J.P., Raguenes G., Guezennec J. (2004). Production of exopolysaccharides by Antarctic marine bacterial isolates. J. Appl. Microbiol..

[9] Nwodo U.U., Green E., Okoh A.I. (2012). Bacterial exopolysaccharides: Functionality and prospects. Int. J. Mol. Sci..

[10] Poli A., Anzelmo G., Nicolaus B. (2010). Bacterial exopolysaccharides from extreme marine habitats: Production, characterization and biological activities. Mar. Drugs.

[11] Liang Z., Yi Y., Guo Y., Wang R., Hu Q., Xiong X. (2014). Chemical Characterization and Antitumor Activities of Polysaccharide Extracted from Ganoderma lucidum. Int. J. Mol. Sci..

[12] Shang N., Xu R., Li P. (2013). Structure characterization of an exopolysaccharide produced by Bifidobacterium animalis RH. Carbohydr. Polym..

[13] Verhoef R., Schols H.A., Blanco A., Siika-Aho M., Rättö M., Buchert J., Lenon G., Voragen A.G.J. (2005). Sugar composition and FT-IR analysis of exopolysaccharides produced by microbial isolates from paper mill slime deposits. Biotechnol. Bioeng..

[14] Pereira L., Alan T., Critchley M.O., Danilo B.L. (2006). Identification of phycocolloids by vibrational spectroscopy. World Seaweed Resources–An Authoritative Reference System.

[15] Pereira L., Gheda F.S., Ribeiro-Claro P.J.A. (2013). Analysis by vibrational spectroscopy of seaweed polysaccharides with potential use in food, pharmaceutical and cosmetic industries. Int. J. Carbohydr. Chem..

[16] Muthukumar A., Baskaralingam V., Mani D., Sekar V., Marimuthu G., Naiyf S.A., Jamal M.K., Mohammed N.A., Giovanni B. (2018). Structural characterization of *Bacillus licheniformis* Dahb1exopolysaccharide—Antimicrobial potential and larvicidal activity on malaria and Zika virus mosquito vectors. Environ. Sci. Pollut. Res..

[17] Koening J.L. (1979). Vibrational Spectroscopy of Carbohydrates. Infrared and Raman Spectroscopy of Biological Molecules.

[18] Mathlouthi M., Koening J.L. (1987). Vibrational spectra of carbohydrates. Adv. Carbohydr. Chem. Biochem..

[19] Wang Z., Sheng J., Tian X., Wu T., Liu W., Shen L. (2011). Optimization of the production of exopolysaccharides by *Bacillus thuringiensis* in sand biological soil crusts and its bioflocculant activity. Afr. J. Microbiol. Res..

[20] Yim J.H., Kim S.J., Ahn S.H., Lee H.K. (2007). Characterization of a novel bioflocculant, p-KG03, from a marine dinoflagellate, *Gyrodinium impudicum* KG03. Bioresour. Technol..

[21] Roberson E.B., Firestone M.K. (1992). Relationship between desiccation and exopolysaccharide production in a soil Pseudomonas sp.. Appl. Environ. Microbiol..

[22] Wu Q.X., Lin D.Q., Yao S.J. (2014). Design of chitosan and its water soluble derivatives-based drug carriers with polyelectrolyte complexes. Mar. Drugs.

[23] Bakos D., Soldan M., Hernandez-Fuentes I. (1999). Hydroxyapatite-collagen-hyaluronic acid composite. Biomaterials.

[24] Kodali V.P., Sen R. (2008). Antioxidant and free radical scavenging activities of an exopolysaccharide from a probiotic bacterium. Biotechnol. J..

[25] Sun L., Wang C., Shi Q., Ma C. (2009). Preparation of different molecular weight polysaccharides from Porphyridium cruentum and their antioxidant activities. Int. J. Biol. Macromol..

[26] Asker M.M.S., Shawky B.T. (2010). Structural characterization and antioxidant activity of an extracellular polysaccharide isolated from *Brevibacterium otitidis* BTS 44. Food Chem..

[27] Papageorgiou S.K., Kouvelos E.P., Favvas E.P., Sapalidis A.A., Romanos G.E., Katsaros F.K. (2010). Metal-carboxylate int eractions in metal-alginate complexes studied with FTIR spectroscopy. Carbohydr. Res..

[28] Patel A., Prajapat J. (2013). Food and health applications of exopolysaccharides produced by lactic acid bacteria. Adv. Dairy Res..

[29] Freitas F., Alves V.D., Reis M.A. (2011). Advances in bacterial exopolysaccharides: From production to biotechnological applications. Trends Biotechnol..

[30] More T.T., Yan S., John R.P., Tyagi R.D., Surampalli R.Y. (2012). Biochemical diversity of the bacterial strains and their biopolymer producing capabilities in wastewater sludge. Bioresour. Technol..

[31] Sun M.L., Zhao F., Shi M., Zhang X.Y., Zhou B.C., Zhang Y.Z., Chen X.L. (2015). Characterization and biotechnological potential analysis of a new exopolysaccharide from the arctic marine bacterium Polaribacter sp. SM1127. Sci. Rep..

[32] Chen L.Y., Du Y.M., Wu H.Q., Xiao L. (2002). Relationship Between Molecular Structure and Moisture-Retention Ability of Carboxymethyl Chitin and Chitosan. J. Appl. Polym. Sci..

[33] Sutherland I.W. (1998). Novel and established applications of microbial polysac-charides. Trends Biotechnol..

[34] El-Zaher E.H.A., Mostafa A.A., El-Souod S.M.A., Enas M. (2015). Optimization and characterization of exopolysaccharides from Pleurotus salmoneo-stramineus and its possible application. Egypt. J. Exp. Biol. (Bot.).

[35] Zhang X.F. (2012). The Screen of Polysaccharide-Producing Marine Bacteria and the Study of Its Polysaccharide Fermentation.

[36] Boyle C.D., Reade A.E. (1983). Characterization of two extracellular polysaccharides from marine bacteria. Appl. Environ. Microbiol..

[37] Park S., Kelley K.A., Vinogradov E., Solinga R., Weidenmaier C., Misawa Y., Lee J.C. (2010). Characterization of the Structure and Biological Functions of a Capsular Polysaccharide Produced by Staphylococcus saprophyticus. J. Bacteriol..

[38] Vincent P., Pignet P., Talmont F., Bozzi L., Fournet B., Guezennec J., Jeanthon C., Prieur D. (1994). Production and characterization of an exopolysaccharide excreted by a deep-sea hydrothermal vent bacterium isolated from the polychaete annelid Alvinella pompejana. Appl. Environ. Microbiol..

[39] Steinmetz I., Rohde M., Brenneke B. (1995). Purification and characterization of an exopolysaccharide of Burkholderia (Pseudomonas) pseudomallei. Infect. Immun..

[40] Lv Y., Yang X.B., Zhao Y., Ruan Y., Yang Y., Wang Z.Z. (2009). Separation and quantification of component monosaccharides of the tea polysaccharides from Gynostemma pentaphyllum by HPLC with indirect UV detection. Food Chem..

[41] Xie J.Z., Zou L.H., Luo X., Qiu L., Wei Q., Luo D., Wu Y.Q., Jiao Y. (2018). Structural characterization and immunomodulating activities of a novel polysaccharide from Nervilia fordii. Int. J. Biol. Macromol..

[42] Fan G., Tang C., Li Y., Yang Y.D., Zhang Y. (2014). Analysis of Monosaccharide Compositions of Polysaccharides in Coptidis Rhizoma by Pre-column Derivatization HPLC Method. Chin. J. Exp. Tradit. Med. Formulae.

[43] Chen Q., Chen J., Du H., Li Q., Chen J., Zhang G., Liu H., Wang J. (2014). Structural characterization and antioxidant activities of polysaccharides extracted from the pulp of Elaeagnus angustifolia L.. Int. J. Mol. Sci..

[44] Marklund S., Marklund G. (1974). Involvement of the superoxide anion radical in the autoxidation of pyrogallol and a convenient assay for superoxide dismutase. Eur. J. Biochem..

